# Case Report: Surgical management of medial collateral ligament calcification

**DOI:** 10.3389/fsurg.2024.1506867

**Published:** 2024-12-02

**Authors:** Yihang You, Zhenhua Li, Jie Guo, Tao Zhang

**Affiliations:** Department of Orthopedics, Jinan Central Hospital, Jinan, Shan Dong, China

**Keywords:** calcification, knee, medial collateral ligament, arthroscopic surgery, minimally invasive

## Abstract

Calcification is a self-limiting disease, characterized by the deposition of calcium, causing severe pain, swelling, and movement disorder. It is mainly found in the shoulder joint but has also been reported in other joints such as the wrist, hip, knee, foot, and ankle. However, calcification of the medial collateral ligament (MCL) has been rarely reported. The patient was a 47-year-old female without any trauma, whose chief complaint was pain and impaired flexion–extension of the affected knee joint. The diagnosis was calcification of the MCL, subsequently demonstrated by imaging examination. Conservative treatment was initially attempted, followed by arthroscopic surgery, and the postoperative pathological results confirmed the calcified tendon. The patient had a favorable prognosis 1 month after the procedure. This study demonstrates that arthroscopic surgery can result in effective and swift recovery of clinical outcomes for patients with calcification of the MCL.

## Introduction

Periarticular calcification is an inflammatory condition associated with calcium hydroxyapatite crystal deposition diseases (1). At present, the cause is unknown, which may be related to factors such as degeneration and strain of the tendon, ischemia and hypoxia, increased sectional pressure, and metabolic abnormalities, such as hyperparathyroidism and other hypercalcemia (2–5). The medial collateral ligament (MCL) is composed of two layers, with the MCL bursa situated between the superficial and deep fibers (6, 7). Calcification has been found either inside the bursa (calcific bursitis) or outside of it. When the bursa is distended by calcific material, it can distend along the proximal direction toward the medial femoral condyle. In general, the material is fluid and smooth, presenting as a polylobed, septate structure in MR analyses (3, 8). Located in the ligament, the calcific deposition is often revealed as a linear or irregular morphology compared with calcific bursitis.

Calcification of the MCL is an uncommon type, confused with meniscal tears, gout, Pellegrini–Stieda syndrome, avulsion fracture, and synovial sarcoma (9, 10). Due to its low incidence rate and the absence of specific performance, clinicians often misdiagnose the rare entity. magnetic resonance imaging (MRI) and x-ray were invaluable tools for the diagnosis.

We aim to enhance the consciousness of joint surgeons to make the precise diagnosis for acute pain and limited mobility of the knee joint. Prompt intervention and effective treatment are crucial in preventing the development of joint stiffness.

## Case report

A 47-year-old woman presented with increasing pain of the right knee associated with limitation of movement for 1 month. There was no history of prior injury, sepsis, or gout nor any other notable family or medical history for any pathological condition. The increasing pain had produced a bad influence on her nighttime sleep and daily activity.

On physical examination, the affected knee joint was slightly swollen without erythema, heat, or effusion. The test revealed a tenderness point in the region of medial condyle of femur. The active range of movements (ROM) of knee was restricted from 0° to 100°, while the passive ROM was from 0° to 110°. Patella grind test was positive. There was no ligament laxity, and clinical tests for detection of meniscus lesions were all negative.

**Figure 1:**
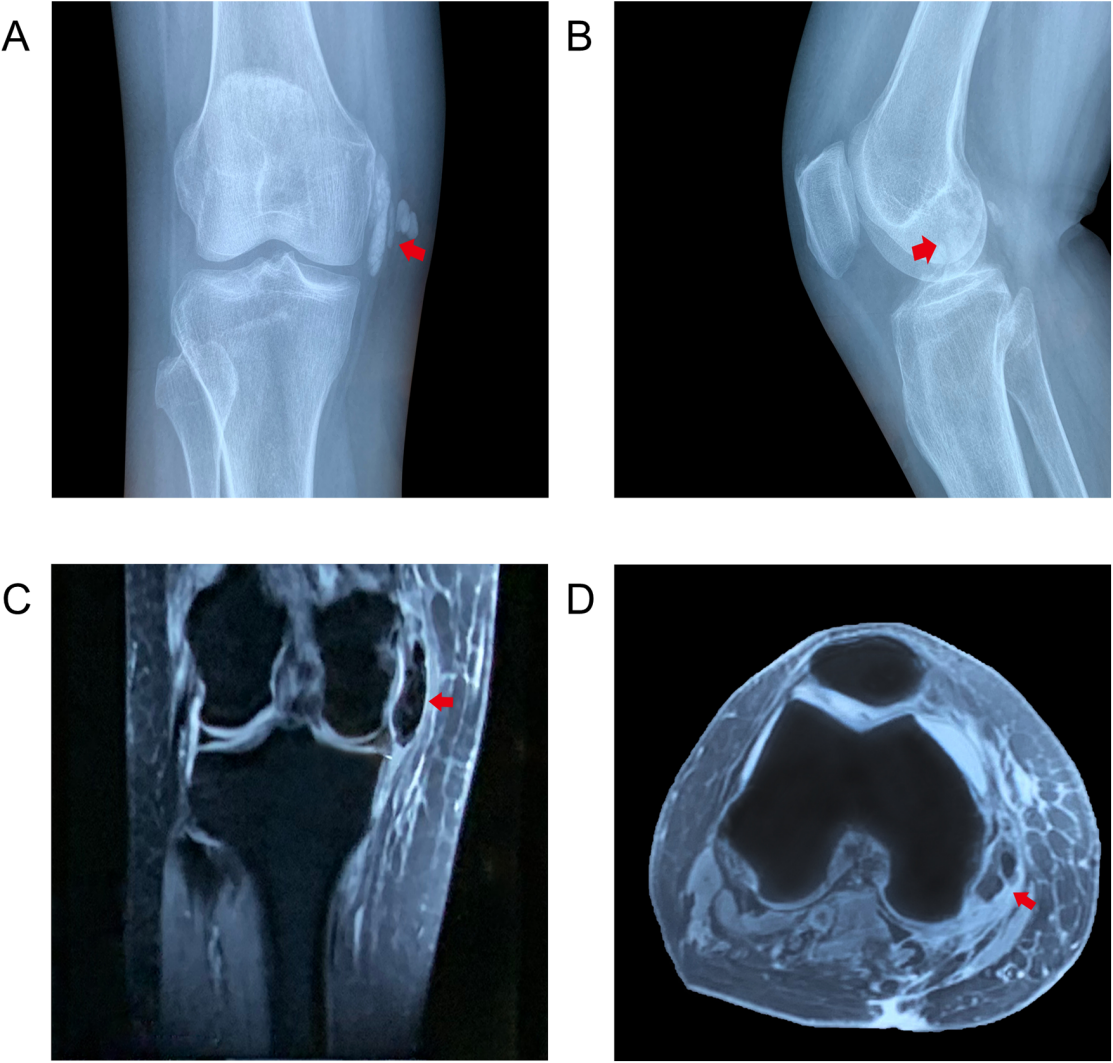
X-ray of right knee joint showed striped dense calcification in the MCL (red arrow): (**A**) coronal and (**B**) sagittal views. MRI showed the calcific mass showing low signal intensity, suggesting calcific deposition in the lateral side of the MCL: (**C**) T1 coronal and (**D**) T1 axial views.

Radiographs revealed a massive striped hyperdense shadow near the medial epicondyle of femur without osteoarthritic changes and evidence of fracture (Figures 1A,B). Meanwhile MRI demonstrated a low signal intensity mass in the lateral side of the MCL (Figures 1C,D). Laboratory examinations, including the routine blood investigations, erythrocyte sedimentation rate (ESR), serum electrolytes test, and C-reactive protein (CRP), were found to be normal.

**Figure 2:**
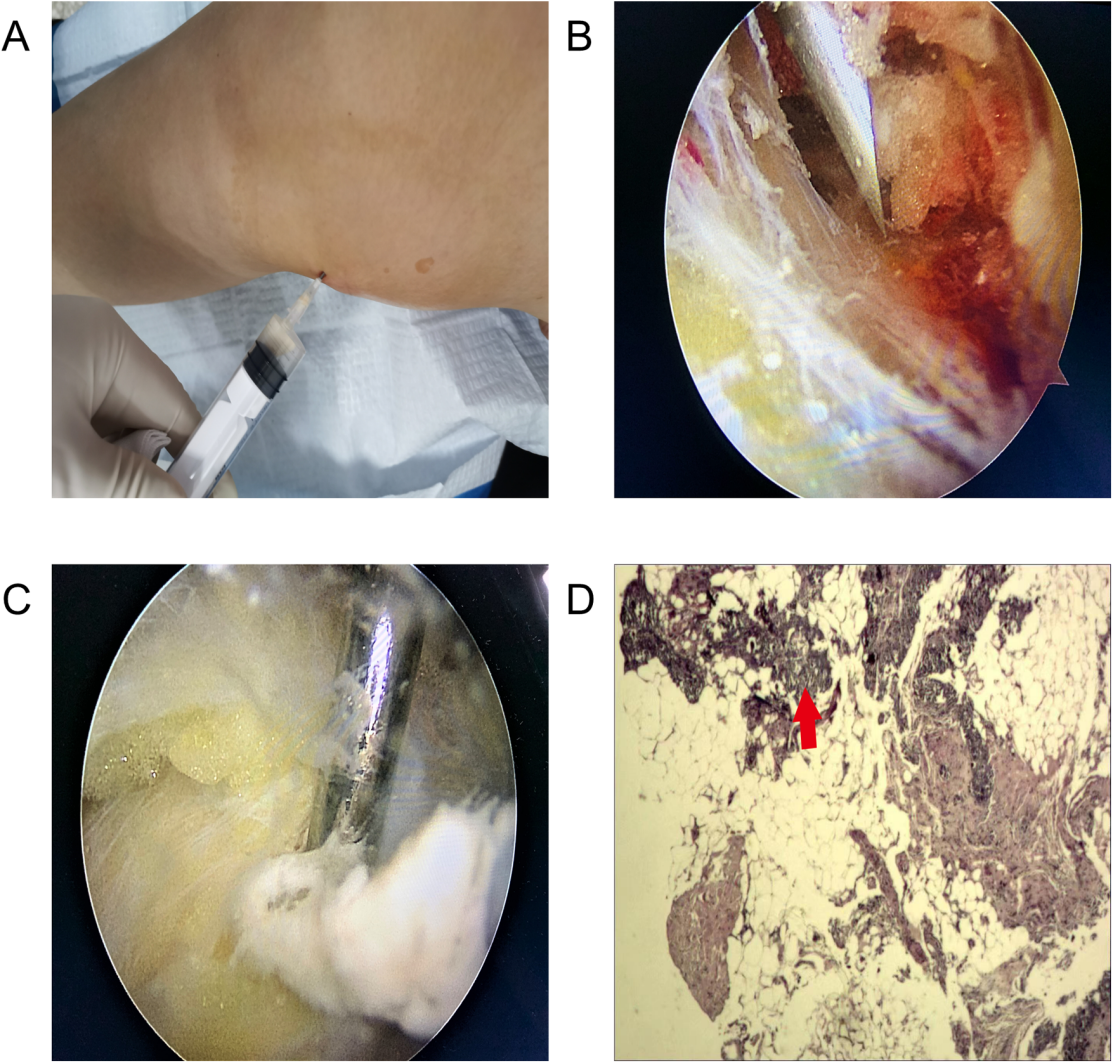
(**A**) Needle aspiration showed some toothpaste-like deposition. (**B**) Arthroscopic views showed synovial congestion and calcific deposition in the MCL. (**C**) Milky, toothpaste-like deposition was excised by using a motorized shaver. (**D**) Histology (HE) showed partial degeneration and calcification in the fibrous connective tissue (red arrow).

In the initial treatment plan, we attempted conservative managements such as needle aspiration of calcium deposits (Figure 2A), corticosteroid injection, and oral administration of non-steroidal anti-inflammatory drugs (NSAIDs). However, she returned in 1 week due to the discomfort and pain in the medial side of the knee joint. As the conservative treatments were inefficient, the patient underwent knee arthroscopy that we recommended.

The patient was placed in supine position after general anesthesia. The pneumatic tourniquet was preset. Arthroscopy revealed strawberry-like synovial congestion between the superficial and deep fascial layers of the MCL. Using a puncture needle to probe, we detected some milky, toothpaste-like lesion flowing out from the tear of the MCL. The calcific deposit region was resected completely from the MCL and sent for pathological examination. We used a motorized shaver, arthroscopic scissors, and electrocautery to exclude the calcification for decompression. The excision of the deposits did not result in defective MCL (Figures 2B,C). Histopathological evaluation revealed the presence of hydroxyapatite crystals (Figure 2D).

Postoperatively, the patient's symptoms such as pain and swelling were significantly alleviated. On the third day, both active and passive ROM of the knee joint were increased that from 0° to 120°. The patients were followed-up 1 month after operation without residual pain and dysfunction.

## Discussion

Calcification can be categorized into three stages: pre-calcification, calcification, and post-calcification (11). The clinical course of the disease fluctuates as it corresponds with the formation and reabsorption of crystals. The pre-calcification stage, in which fibrocartilaginous transformation takes place within the tendon fibers, is usually asymptomatic. The calcific stage is characterized by a formative phase, typically involving sub-acute and low-grade pain that intensifies during the night. This is followed by a quiescent phase and a resorptive phase, during which the calcium salt deposit manifests in the form of cream or toothpaste, occasionally causing severe pain (12). In the post-calcific stage, the symptoms are generally significantly reduced or gradually alleviated. However, the aforementioned clinical features lack specificity.

For most patients with calcification, conservative treatment for 4–6 weeks can achieve cure or remission such as observation, physiotherapy, local injections, and ultrasonic shockwave therapy. Yamamoto et al. (13) pointed out that H2 blockers can inhibit the calcification progress of calcified tendinitis and can be used to relieve pain and treat tendon ossification or calcification. Ultrasound guidance is not only a valuable method to release local adhesions by hydrodissection or drug injection but also a first-line approach to treat the calcific bursitis of the MCL (14). The selection of therapeutic schedule depends on the anatomical localizations of the calcium deposits and on the clinical conditions of the patient. However, in many cases, conservative treatments for tendon calcification are ineffective, necessitating surgery for the patients. White et al. (11) described an open procedure for the removal of calcified lesions, considering operative excision may provide immediate pain relief. Kamawal et al. (15) found that calcification of the MCL was more likely to require surgery than calcification of the rotator cuff. Since the advent of minimally invasive theory, arthroscopic surgery is gradually regarded as the top choice for treating calcification of the MCL. The treatments for MCL calcification are summarized in Table 1.

**Table 1:** Review of articles on the treatment of calcification of the MCL.

No.	First author	Patient no.	Treatment	Follow-up	Outcomes	Reference
1	Mansfield HL	1	Conservative approaches as taking NSAIDs	2 months	Pain-free	(16)
2	Song K	1	Arthroscopic excision of calcific deposit	1 month	Recovery	(17)
3	White WJ	1	Operative excision	6 months	Recovery	(11)
4	Kamawal Y	1	An arthroscopy followed by an open procedure	4 weeks	Pain-free	(15)
5	Castillo-González F	1	Ultrasound-guided percutaneous lavage	1 month	Symptom-free	(8)
6	Galletti L	1	Double-needle ultrasound-guided percutaneous lavage	4 weeks	Symptom-free	(3)

After unsuccessful attempts via needle aspiration, corticosteroid injection, and oral administration of NSAIDs, arthroscopic surgery was employed to treat the patient in our case, as a minimally invasive method. For patients with calcification of the MCL characterized by an acute onset, severe symptoms, non-responsive conservative measures, recurrent attacks, and large lesion sizes, arthroscopic lesion clearance is now frequently recommended, alleviating symptoms immediately and allowing early rehabilitation postoperatively. Furthermore, it is essential to bear in mind that obtaining a biopsy to confirm the diagnosis is crucial.

## Conclusion

This case report illustrates that arthroscopic treatment is a better choice for calcification of the MCL with the advantage of providing the required outcome if conservative treatment fails. During the operation, removing the calcification completely and sampling to perform the pathological diagnosis are important. Scientific inferences must be supported by independent data. However, the current study involves no data to compare the superiority of the conservative treatment against surgical treatment. Therefore, a prolonged follow-up investigation and a larger sample of patients are required to establish reliable conclusions.

## Data Availability

The original contributions presented in the study are included in the article/Supplementary Material, further inquiries can be directed to the corresponding author.
